# High-Throughput 3D *In Vitro* Tumor Vasculature Model for Real-Time Monitoring of Immune Cell Infiltration and Cytotoxicity

**DOI:** 10.3389/fimmu.2021.733317

**Published:** 2021-09-24

**Authors:** Jiyoung Song, Hyeri Choi, Seung Kwon Koh, Dohyun Park, James Yu, Habin Kang, Youngtaek Kim, Duck Cho, Noo Li Jeon

**Affiliations:** ^1^ Department of Mechanical Engineering, Seoul National University, Seoul, South Korea; ^2^ Interdisciplinary Program in Bioengineering, Seoul National University, Seoul, South Korea; ^3^ Department of Health Sciences and Technology, Samsung Advanced Institute for Health Sciences & Technology (SAIHST), Sungkyunkwan University, Seoul, South Korea; ^4^ Department of Laboratory Medicine and Genetics, Samsung Medical Center, Sungkyunkwan University School of Medicine, Seoul, South Korea; ^5^ Institute of Advanced Machines and Design (SNU-IAMD), Seoul National University, Seoul, South Korea

**Keywords:** tumor vasculature, microfluidics, live-cell imaging, adoptive cell therapy, NK cell cytotoxicity

## Abstract

Recent advances in anticancer therapy have shown dramatic improvements in clinical outcomes, and adoptive cell therapy has emerged as a type of immunotherapy that can modulate immune responses by transferring engineered immune cells. However, a small percentage of responders and their toxicity remain as challenges. Three-dimensional (3D) *in vitro* models of the tumor microenvironment (TME) have the potential to provide a platform for assessing and predicting responses to therapy. This paper describes an *in vitro* 3D tumor model that incorporates clusters of colorectal cancer (CRC) cells around perfusable vascular networks to validate immune-cell-mediated cytotoxicity against cancer cells. The platform is based on an injection-molded 3D co-culture model and composed of 28 microwells where separate identical vascularized cancer models can be formed. It allows robust hydrogel patterning for 3D culture that enables high-throughput experimentation. The uniformity of the devices resulted in reproducible experiments that allowed 10× more experiments to be performed when compared to conventional polydimethylsiloxane (PDMS)-based microfluidic devices. To demonstrate its capability, primary natural killer (NK) cells were introduced into the vascularized tumor network, and their activities were monitored using live-cell imaging. Extravasation, migration, and cytotoxic activity against six types of CRC cell lines were tested and compared. The consensus molecular subtypes (CMS) of CRC with distinct immune responses resulted in the highest NK cell cytotoxicity against CMS1 cancer cells. These results show the potential of our vascularized tumor model for understanding various steps involved in the immune response for the assessment of adoptive cell therapy.

## Introduction

Cancer treatment has evolved over the years from traditional chemotherapy to targeted therapy and cancer immunotherapy to minimize the systemic toxicity of anticancer drugs (1). Cancer immunotherapy holds great potential as an effective treatment, offering fewer side effects by modulating the host immune system (2). In addition to checkpoint inhibitors, adoptive cell transfer of engineered T cells or natural killer (NK) cells is another emerging approach; however, only a small number of patients with specific cancer types show beneficial responses to immunotherapy thus far (3, 4). Plenty of traditional *in vitro* cancer models based on two-dimensional (2D) culture systems or animal models have accelerated the development of anticancer therapies (5, 6), yet the limitations of the models remain concerning the absence of the main components in the tumor microenvironment (TME), and species-dependent differences (7, 8).

*In vitro* three-dimensional (3D) models for the study of tumor–immune cell interaction have been proposed while excluding certain components in the TME (9–12). For example, a previous study by our group introduced a microfluidic device for NK cell cytotoxicity assays against HeLa cells in a 3D avascular environment (13). Modeling tumor vasculature is considered essential, since cancer cells interact with surrounding vascular networks, which provide a continuous supply of oxygen and nutrients to tumor tissues and promote tumor progression (14). Aberrant tumor vasculature completely differs from normal vasculature and has distinct features such as a chaotic flow pattern or high permeability. This disorganized and immature vasculature also hinders anticancer drugs or immune cells from targeting tumor cells, resulting in reduced success rates of therapies (15). Inhibiting angiogenesis by targeting proangiogenic factors such as vascular endothelial growth factors (VEGFs) is a promising therapeutic approach to normalize blood vessels around a tumor (16). However, the consequence of reduced angiogenesis may induce responses of cancer cells to hypoxia, which makes cancer cells more aggressive, and the normalized tumor vasculature is no longer an accurate pathway for drug delivery (17). These controversial debates on the role of tumor vasculature in cancer therapy have been ongoing research topics that can be resolved by *in vitro* studies (14). Several recent studies using microfluidic devices have been proposed to recapitulate the TME with perfusable vessels; however, their low-throughput and laborious experimental design including an external fluid pump make it impractical to use for clinical purposes (18–21). Thus, there is an urgent need for improved *in vitro* tumor vasculature models including the complex interplay between tumors and their surrounding microenvironment.

Herein, we developed a perfusable 3D tumor vasculature model on a high-throughput microfluidic platform for a better understanding of immune cell infiltration and cytotoxicity against cancer cells. By altering cancer cell seeding density and the medium composition through high-throughput experimentation, we optimized the culture condition for constructing tumor vasculature, and a vascular permeability assay was performed to ensure that immune cells were later allowed to travel *via* the vascular networks and migrate toward cancer cells. Additionally, we evaluated the cytotoxic activity of NK cells against colorectal cancer (CRC) cell lines derived from different consensus molecular subtypes (CMS), which categorizes CRC into four transcriptome-based subtypes (22), to demonstrate one of the possible applications using our platform. The results showed the potential of our platform in adoptive cell therapy, since perfusable vascular networks in the model provided a route for the transfer of immune cells from a distant site to the tumor site. We expect that our platform can be considered a powerful tool for studying tumor–immune cell interactions and for efficacy testing and drug screening in the preclinical stages.

## Materials and Methods

### Microfluidic Platform Fabrication

The microfluidic platform was fabricated by polystyrene injection molding at R&D factory (Korea). This process involved machining and polishing steps for an aluminum alloy mold, and injection molding was performed with a clamping force of 130 tons and 55 bar injection pressure for 15 s, while the nozzle temperature was 220°C. The injection-molded microfluidic platforms underwent surface oxygen plasma treatment at 5.00e−1 Torr and 50 W for 3 min and were then bonded to a pressure sensitive adhesive film.

### Cell Culture

Human umbilical endothelial cells (HUVECs; Lonza, Switzerland) were cultured in endothelial growth medium 2 (EGM-2; Lonza, Switzerland), and passages 4 and 5 were used. Lung fibroblasts (LFs; Lonza, Switzerland) were cultured in fibroblast growth medium 2 (FGM-2; Lonza, Switzerland), and passages 6 and 7 were used. All CRC cell lines were cultured in Roswell Park Memorial Institute (RPMI)-1640 medium (Gibco, USA) supplemented with 10% fetal bovine serum (FBS; HyClone, USA) and 1% penicillin-streptomycin (PS; Gibco, USA). Primary NK cells were expanded from peripheral blood mononuclear cells (PBMCs) by co-culturing with 100 Gy-irradiated K562-OX40L-membrane-bound (mb) IL-18/21 feeder cells (23). In particular, human PBMCs were isolated from healthy adult donors using density-gradient centrifugation with Ficoll–Hypaque (d = 1.077, LymphoprepTM; Axis-Shield, Oslo, Norway) and washed twice with phosphate-buffered saline (PBS) (Welgene, USA). PBMCs were co-cultured with feeder cell (6:1 ratio) in a 24-well-plate with RPMI 1640 culture medium supplemented with 10% fetal bovine serum (FBS) (Gibco), 100 U/ml penicillin, 100 μg/ml streptomycin, and 4 mmol/L L-glutamine (Thermo Fisher) containing 10 U/ml recombinant human IL-2 (Peprotech). After 1 week of culture, the concentration of IL-2 was increased to 100 U/ml, and 5 ng/ml of soluble IL-15 (Peprotech) was added to the fresh medium to induce vigorous proliferation. The medium was replaced every 2–3 days. Expanded NK cells from day 14–21 were cryopreserved and used for NK cell killing assay with live imaging when needed. No data were used for personal identification of human PBMC. The use of human primary NK cells was approved by the Institutional Review Board in Samsung Medical Center (No. SMC 2020-08-033-002).

### Cell Seeding

HUVECs, LFs, and CRC cells were resuspended in EGM-2 and mixed with fibrinogen solution (final concentration of 2.5 mg/ml; Sigma-Aldrich, USA) and bovine thrombin (0.5 U/ml, Sigma-Aldrich, USA) immediately before cell seeding on the platform. The final cell concentrations of HUVECs, LFs, and CRC cells were 6 × 10^6^ cells ml^−1^, 2 × 10^6^ cells ml^−1^, and 0.1 to 0.3 × 10^6^ cells ml^−1^, respectively. Of this acellular mixture, 0.9 µl was injected into the central channel and incubated for 6 min for cross-linking. Then, the side channel was filled with 3 µl of HUVEC suspension at a concentration of 3 × 10^6^ cells ml^−1^, and the platform was tilted 90° for 15 min to allow HUVECs to cover the side of the fibrin gel. This process is repeated for the opposite side channel. The medium reservoirs were filled with 200 µl of EGM-2 supplemented with 10% or 20% FBS, and the platform was incubated at 37°C with 5% CO_2_. The medium was changed every day for 5 days. The experiment under the same culture conditions were repeated twice on different days, and a total of 14 experimental samples were collected for each cancer cell line from every experiment.

### Immunocytochemistry

The cells were fixed with 4% (w/v) paraformaldehyde (Biosesang, Korea) for 15 min and permeabilized with 0.2% Triton X-100 (Sigma-Aldrich, USA) for 15 min. EC-specific staining was performed using fluorescein-labeled Ulex Europaeus Agglutinin I (1:1,000; Vector, UK) and DyLight 594-conjugated Ulex Europaeus Agglutinin I (1:1,000; Vector, UK). Alexa Fluor 488 tagged antiepithelial cell adhesion molecule (EpCAM; Biolegend, USA) was used at a dilution of 1:200 in bovine serum albumin (BSA) for tumor-specific surface staining. Unlike other CRC cell lines, EC-specific staining used in this experiment was also positive in LoVo cells. Their merged images (green and red) show co-stained sections in LoVo cells in yellow. All the samples were maintained in PBS at 4°C until imaging.

### Vascular Permeability Coefficient

Vascular permeability was measured at day 5. Firmly perfusable vessels were labeled with lectin (red, 1:2,000 dilution; Vector, UK), and CRC cells were labeled with EpCAM (green, 1:500 dilution; Biolegend, USA) before measurement. Regions of interest (ROIs) were selected based on the regions where both perivascular and intravascular regions were evenly distributed. The cell culture medium was removed from each well, and a solution containing fluorescence day was added to the medium reservoirs. Cascade blue (MW = 3 kDa) was used here to measure the endothelial barrier permeability. The samples were imaged every 20 s for 10 min in a live-cell imaging chamber. The vascular permeability coefficient was calculated based on the equation derived from previous research, and the final value was the average of six samples for each condition.

### Live-Cell Imaging

Time-lapse live-cell imaging was performed using a Ti2-Eclipse inverted microscope with NIS elements software (Nikon, Japan). After immunofluorescence staining of HUVECs and CRC cells for at least 3 h prior to imaging, NK cells were labeled with CellTrace™ Far Red Cell Proliferation Kit (Thermo Fisher, C34572) at a dilution of 1:1,000 in serum-free RPMI-1646 medium for 30-min incubation. The same amount of serum-included RPMI-1646 medium was added to the serum-free RPMI-1642 medium and incubated for another 5 min. The concentrations of NK cells at 0.05 × 10^6^ cells ml^−1^ and 0.1 × 10^6^ cells ml^−1^ were used for NK cell cytotoxicity assays. Once NK cells were introduced into the vessels by gravity-driven flow, time-lapse live-cell imaging was performed for 24 h, while the samples were maintained in a live-cell imaging chamber at 37°C with 5% CO_2_.

### Image Analysis

Confocal images were analyzed using Image J. After 3D images were converted to 2D images by z-projection, the region of interest was cropped and converted to a binary mask obtained using thresholding. All image-based data analysis including the total areas of blood vessels and cancer cells was performed automatically, and the number of junctions in tumor vasculature was analyzed using Angiotool (National Cancer Institute).

### Statistical Analysis

Statistical comparisons were performed using GraphPad Prism software. Ordinary one-way ANOVA with multiple comparisons was used to obtain the statistical value. The p-value thresholds for statistical significance were set: *p < 0.1. **p < 0.01; ***p < 0.001; ****p < 0.0001; and ns (not significant). The standard error of the mean was presented in error bars.

## Results

### Tumor Vasculature Model on the High-Throughput Platform

**Figure 1:**
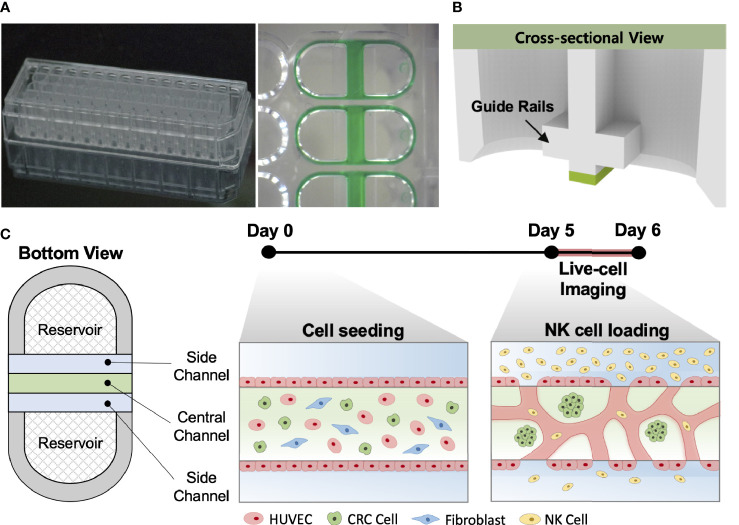
*In vitro* tumor vasculature model on the injection-molded microfluidic platform. **(A)** Photograph of the injection-molded microfluidic platform. **(B)** Illustration of cross-sectional view of hydrogel patterning on the platform. The platform is composed of 3 parallel fluid guide rails, and rapid and robust fluid patterning driven by capillary forces enables high-throughput experiments. **(C)** Cell configuration in tbe tumor vasculature model and experiment time from cell seeding (Day 0) to live-cell imaging (Day 5-6). Perfusable blood vessel networks with CRC cell clusters can be formed within 5 days. To demonstrate the capability of this model, primary NK cells were introduced into the vascular network, and their migration, extravasation, and killing were monitored by live-cell imaging. Scale bar = 200µm.

Our injection-molded platform comprising 28 wells was designed for a straightforward hydrogel patterning technique and a high degree of compatibility with general laboratory equipment. The platform provides rapid and robust hydrogel patterning, which takes approximately 30 s to complete the entire row with a high success rate (**Figure 1A**). These characteristics allow the platform to be suitable for high-throughput experimentation with a variety of applications (24). The platform has three parallel microchannels as fluid guide rails designed to pattern different types of hydrogels or cell suspensions. The rails work as guiding structures, which allow fluid to flow spontaneously into the channels due to capillary forces produced on hydrophilic surfaces (**Figure 1B**) (25). The mixture of HUVECs, LFs, and CRC cells with the hydrogel solution was injected into the central channel and cultured for 5 days until constructing firmly perfusable vessels. NK cells were then introduced into the vessels through the side channel, and cellular responses in the model were monitored by time-lapse live-cell imaging for 24 h (**Figure 1C**).

### Optimization of Cell Culture Media for *In Vitro* Tumor Vasculature

**Figure 2:**
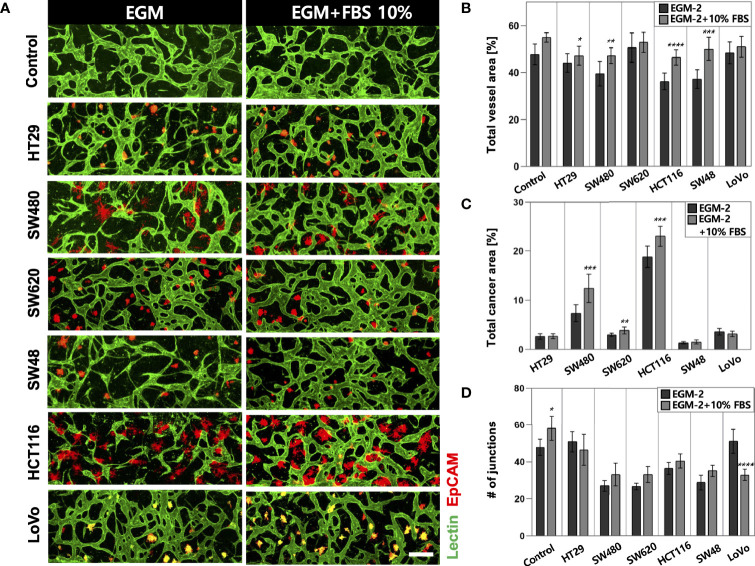
Optimization of the culture condition for the formation of perfusable blood vessels co-cultured with CRC cells. **(A)** Confocal images of blood vessels (lectin, green) with various types of CRC cells (EpCAM, red) in EGM-2 or EGM-2 supplemented with 10% FBS medium. **(B)** Plot of total vessel area for different CRC cell lines. Total vessel area increased when 10% FBS was added to the culture medium. **(C)** Plot of total cancer cell area for various CRC cell lines. The cancer area was significantly increased in some CRC cell lines (SW480, SW620, and HCT116) in EGM-2 supplemented with 10% FBS. **(D)** Plot of the number of junctions in the vascular network. The junction numbers are mostly higher in EGM-2 supplemented with 10% FBS except for the vessels co-cultured with HT29 and LoVo (n = 14 for each cancer cell line). *p < 0.1; **p < 0.01; ***p < 0.001; ****p < 0.0001. Scale bar = 200µm.

High-throughput experimentation using our platform allowed us to quickly determine the optimal conditions for culturing *in vitro* tumor vasculature, each induced by CRC cell lines with distinct subtypes (**Supplementary Table S1**). The sufficient amount of additional FBS in the culture media could satisfy both CRC cells and ECs to construct robust blood vessels (**Figure 2A**). The total areas of blood vessels and cancer regions were increased in all CRC cell lines when the serum concentration increased to 10% (**Figure 2B**). HT29, SW480, HCT116, and SW48 showed significant changes in the blood vessel area, which were increased by 3.09%, 7.71%, 10.43%, and 12.85%, respectively. There was also an increasing tendency of the vessel area when the serum concentration in the culture medium increased to 20% (**Supplementary Figures S1A–C**). SW480 and HCT116 showed increased total cancer areas of 5.03% and 4.57%, indicating higher proliferation rates compared to the other CRC cell lines (**Figure 2C**). Since single cancer cells in the TME continuously proliferate and form individual clusters as they grow, this result might come not only from differences in proliferation rates but also their distinctive ways to form clusters (**Supplementary Figures S2A–C**). Additionally, the number of junctions indicated connections between the branches and was higher in most cases under the condition with additional 10% FBS (**Figure 2D**). EGM-2 supplemented with 10% FBS was used as the optimal cell culture media for the rest of our experiments to obtain high reproducible and perfusable tumor vasculature in our model.

### Optimization of Cancer Cell Concentration for *In Vitro* Tumor Vasculature

**Figure 3:**
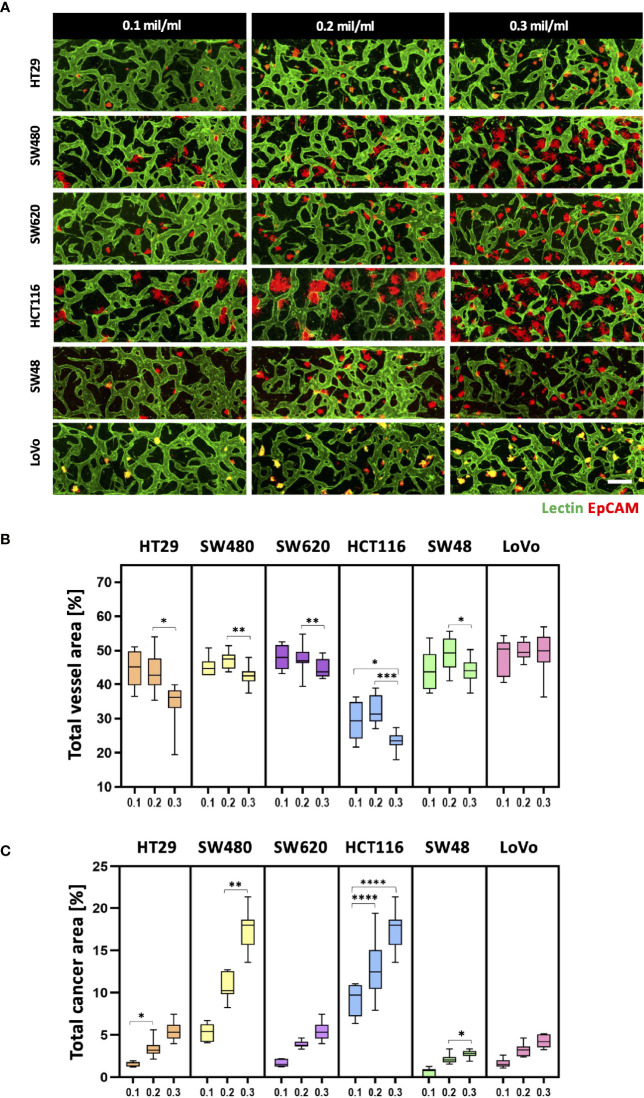
Optimization of CRC cell concentration for the vascularized tumor model. **(A)** 3D confocal images of blood vessels (lectin, green) co-cultured with CRC cells (EpCAM, red) at three different cancer cell concentrations. **(B)** Total vessel area for each cancer cell concentration. The cancer cell concentration at 0.2 × 10^6^ cells mL^-1^ was considered the optimal condition for constructing perfusable blood vessel networks in our model while preserving sufficient cancer cell cluster sizes. **(C)** Plot of total cancer area at different CRC cell concentrations. SW480 and HCT116 showed a dramatic increase in total cancer area when cancer cells were initially seeded at high concentrations (n = 14 for each cancer cell line). *p < 0.1; **p < 0.01; ***p < 0.001; ****p < 0.0001. Scale bar = 200 µm.

Since cancer cells produce a lot of metabolic wastes such as lactic acid (26), cancer cell concentration should be optimized in order to preserve the formation of blood vessels in a microscale channel. We first tested cancer cell concentrations ranging from 0.1 × 10^6^ to 0.3 × 10^6^ cells ml^−1^, considering high proliferation rates of cancer cells (**Figure 3A**). As a result, there was a small difference in the total vessel area when the cancer cell concentration increased from 0.1 × 10^6^ to 0.2 × 10^6^ cells ml^−1^. SW480 and HCT116 have vessel areas increased by 1.94% and 2.75%, respectively, at the cancer cell concentration of 0.2 × 10^6^ cells ml^−1^ (**Figure 3B**). On the other hand, the total vessel area decreased in most CRC cell lines when the cancer cell concentration was at 0.3 × 10^6^ cells ml^−1^. Especially, SW480 and HCT116 showed approximately 4.69% and 9.00% decreases in the total vessel area compared to the cancer cell concentration of 0.2 × 10^6^ cells ml^−1^. The total cancer area gradually increased when cancer cells were initially seeded at high concentrations, and there was a dramatic increase in SW480 and HCT116 (**Figure 3C**). This result can indicate either the proangiogenic effect of CRC cells or the adverse effects of acidic wastes on blood vessels. We hypothesized that growth factors and cytokines such as VEGF-A secreted from cancer cells enhanced angiogenic sprouting (27), whereas excess acidic wastes accumulated in the culture medium overwhelmed the formation of new blood vessels within our platform (28). In other words, the effect of proangiogenic factors could be dominant up to a certain point, and thereafter effects of wastes secreted from cancer cells prevailed; this hypothesis was not confirmed in this study since our goal here was to maximize cancer cell cluster sizes and thoroughly preserve blood vessels in our model. The cancer cell concentration of 0.2 × 10^6^ cells ml^−1^ was used as our optimal cell culture condition.

### Differences in the Size of Cancer Cell Clusters and Vascular Permeability Between CRC Cell Lines

**Figure 4:**
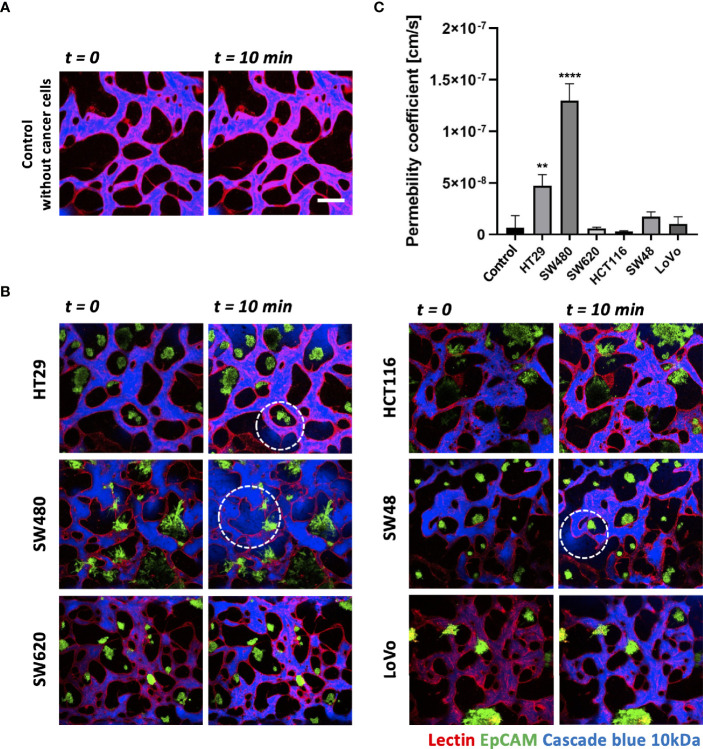
Vascular permeability of normal and tumor vasculature. **(A)** Time-lapse 3D confocal images of normal vasculature. Cascade blue (MW = 3kD) was used to measure vascular permeability. **(B)** Time-lapse 3D confocal images of tumor vasculature. Blood vessels and CRC cells were labeled with lectin (red) and EpCAM (green), respectively. The blue fluorescent dye leaked from weak tight junctions on the vascular wall marked by a white dashed circle. **(C)** Plot of relative permeability coefficients. HT29 and SW480 were more permeable than other conditions (n = 6 for each condition). **p < 0.01; ****p < 0.0001. Scale bar = 200 µm.

Each CRC cell line co-cultured with HUVECs and LFs formed morphologically and functionally different TMEs at day 5 after cell seeding on the platform. CRC cell lines have different proliferation rates and form unique aggregate shapes, which vary from spherical to star-like shapes (29). We determined the size of individual cancer cell clusters and observed that HT29, SW620, and SW48 formed relatively small clusters in near-spherical shapes (**Supplementary Figures S2A, B**). Even though the concentration of FBS in the culture medium increased to 20%, there were no significant differences in the distribution of the cluster sizes (**Supplementary Figure S2C**). HCT116 and SW480 formed wide and sparse star-shaped aggregates, and their sizes are larger than those of other CRC cell lines. The interaction between the size of cancer cell clusters and lymphocyte extravasation will be briefly discussed later in this paper on whether larger cancer cell clusters get more chances to induce lymphocyte extravasation from peripheral blood vessels to tumor sites. In addition, we evaluated vascular permeability coefficients of tumor vasculature co-cultured with each CRC cell line. **Figures 4A, B** show time-series confocal images of tumor vasculature, and cascade blue as a fluorescent dye was used to analyze intensity changes in perivascular regions for permeability measurement. The fluorescence intensity slightly increased at the perivascular region due to fluorescence dye leakage from blood vessel barriers. The permeability coefficient P was calculated based on the equation below:


$$P=\frac{1}{I_{w}}\times \frac{dI/dt}{I_{j}}$$

where *I_w_* is the length of the vessel wall that separates the perivascular and intravascular regions, *I_j_* is the mean intensity in the intravascular region, and I is the total intensity in the perivascular region. More detailed derivation of this equation is described in our previous research (30). Fluorescence dyes started to leak from weak tight junctions of the vessels, marked by dashed white circles (**Figure 4B**). A total of six vascularized tumor models for each CRC cell line were used for quantitative analysis, and **Figure 4C** shows their relative permeability coefficients. Blood vessels induced by HT29 and SW480 are more hyperpermeable compared to normal vasculature without cancer cells and other cell lines, demonstrating differences in vascular permeability between CRC cell lines.

### Validation of NK Cell Infiltration and Cytotoxicity in the Complex 3D TME

**Figure 5:**
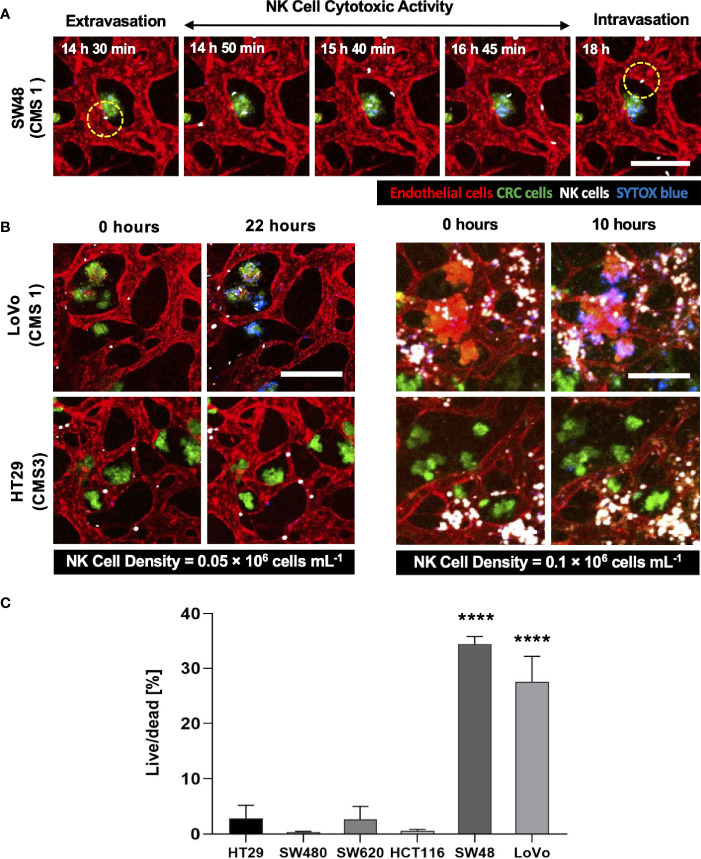
3D confocal images of NK cell cytotoxicity against CRC cells. **(A)** Time-lapse live-cell images of NK cell extravasation, migration, and their cytotoxic activity. Live-cell imaging was performed for 24 h. **(B)** Validation of NK cell cytotoxicity at relatively low and high concentrations of primary NK cells. NK cells (white) showed higher cytotoxic activity against LoVo (CMS1) compared to HT29 (CMS3). As the number of NK cells increased, more CRC cells were killed in a shorter period of time. **(C)** Live/dead assay on 6 different CRC cell lines. The result showed different cytotoxic effects of NK cells against CRC cells. See also **Supplementary Videos S1** **–** **S6** (n = 4 for each CRC cell line). ****p < 0.0001. Scale bar = 200µm.

**Figure 6:**
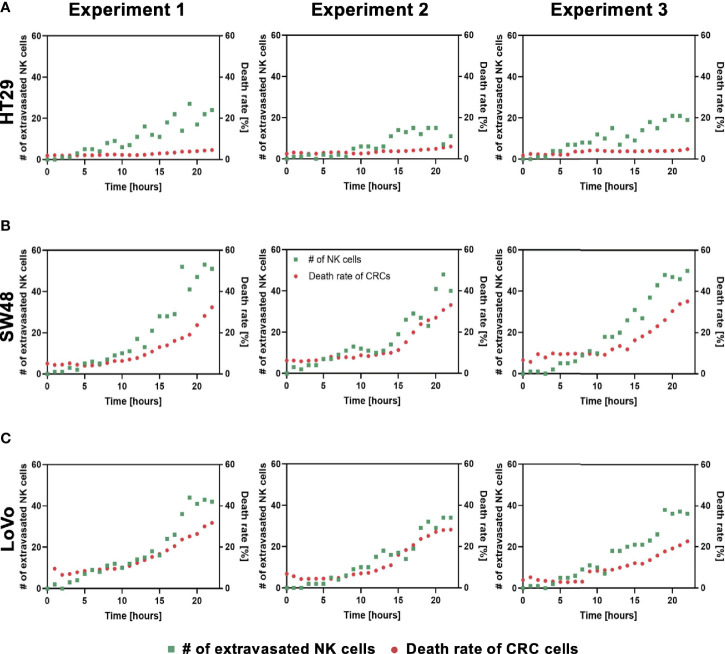
Interactions between NK cell extravasation and their cytotoxic effects. **(A)** The number of NK cell extravasation (green) plotted with the number of cancer cells killed by NK cells (red) over time. The death rate of CRC cells showed no significant changes in HT29 (CMS3). **(B)** SW48 and **(C)** LoVo showed exponential increases in both the number of extravasated NK cells and the death rate of CRC cells. (n = 3 for each CRC cell line).

Using our tumor vasculature model, we demonstrated NK cell cytotoxicity assays according to the subtypes of CRC. Since cytotoxic lymphocytes such as NK cells play a critical role in immune surveillance and continuously circulate in the blood to monitor and destroy cancer cells (31), *in vitro* validation of their cytotoxicity should be performed in the complex 3D TME including blood vessels. We compared the cytotoxicity of primary NK cells on a conventional 2D cell culture dish to our 3D culture platform. Primary NK cells used in our assays were expanded from PBMCs using genetically engineered feeder cells developed in previous research (23). After NK cells were added to the 2D cell culture dish, most cancer cells were dead within 4 h (**Supplementary Figure S3A**), while NK cells in our 3D tumor vasculature model had relatively slower cytotoxic effects, which involved additional processes including NK cell extravasation and intravasation (**Supplementary Figures S3B–D**). NK cells migrated toward the tumor site across the vascular barrier and killed CRC cells, and it took approximately 3.5 h from extravasation to intravasation in this assay (**Figure 5A**). Then, we observed distinct cytotoxic activities of NK cells according to the CMS classification. CRC cell lines with CMS1, CMS3, and CMS4 were used in this assay (**Supplementary Table S1**). Blue fluorescence indicating dead cells appeared on cancer cells immediately after the attacks by NK cells, and the cytotoxic effect of NK cells was barely observed toward endothelial cells and fibroblasts (**Figure 5B** and **Supplementary Videos S1**–**S6**). As a result, NK cells showed much higher cytotoxic activity against LoVo and SW48 (CMS1) compared with other CRC cell lines. There were no significant differences between CMS3 and CMS4 CRC cell lines in the cytotoxic activity. SW620 slightly induced NK cell cytotoxicity; however, a notable result was not found (**Figures 5B, C**). In addition, we used 0.05 × 10^6^ cells ml^-1^ and 0.1 × 10^6^ cells ml^−1^ of NK cells to validate their cytotoxicity according to the ratio of NK cells to cancer cells (**Figure 5B**). Under the condition with the concentration of 0.05 × 10^6^ cells ml^−1^, LoVo cells were mostly killed after 24 h (**Figure 5B**), while NK cells at the concentration of 0.1 × 10^6^ cells ml^−1^ required a relatively short time (i.e., <10 h) to eliminate LoVo cells; however, their cytotoxicity against HT29 remained low (**Figure 5B**). This result shows that a high ratio of NK cells to cancer cells provides more rapid cytotoxic responses to cancer cell, but the trends of NK cell cytotoxicity against different subtypes of CRC cells are similar regardless of the ratio. Intriguingly, among the number of extravasated NK cells, not all NK cells were involved in the cytotoxic activity; only a low number of NK cells resulted in the cytotoxic effect and low viability in cancer cells. The number of extravasated NK cells is plotted with the number of cancer cells killed by NK cells for HT29 (CMS3), SW48, and LoVo (CMS1) (**Figures 6A–C**). The results confirmed that CRC cells with CMS3 induce fewer chances of NK cell extravasation compared with other subtypes, and even though NK cells extravasated into the extracellular matrix around CRC cells, their killing capacity varied with the subtypes and resulted in different cytotoxic effects of NK cells against CRC cells as shown in the time period between 10 and 20 h on the graph. Both the number of extravasated NK cells and the death rate of CRC cells for SW48 and LoVo increased exponentially, but in the case of HT29, the number of extravasated NK cells slightly increased over time, while the death rate of CRC cells showed no significant changes (**Figures 6A–C**).

## Discussion

There are typical characteristics of tumor vasculature: variable vessel diameters, chaotic flow patterns, and high permeability resulting in barrier function defection (32). These features are crucial in both conventional chemotherapies and the latest anticancer immunotherapies since the abnormal form of tumor vasculature hinders the blood circulation around tumors and eventually obstructs drug delivery (33). We focused on defining adequate conditions for a standard *in vitro* tumor vasculature model to study the TME itself and the effects of anticancer drugs or immune cells on tumors. When culturing tumor cells *in vitro*, acidic wastes such as lactate are accumulated in the TME due to cancer cell metabolism (34), and these excess wastes may provide a harsh acidic environment for ECs to be vascularized on our platform due to limited medium volume in the reservoirs. Therefore, the optimal medium condition and cancer cell concentration for tumor vasculature should be determined to compensate for nutrient consumption and to eliminate acidic wastes. There is one major reason for considering the addition of serum to the culture medium. Most cancer cells are cultured in Dulbecco’s modified Eagle’s medium (DMEM) or RPMI supplemented with 10% FBS, while EGM-2 consists of 2% FBS, possibly resulting in a lack of sufficient nutrients in the aspect of ECs when co-cultured with rapidly proliferating cancer cells in EGM-2. Besides, we wanted to maximize the size of cancer cell clusters located near blood vessels in order to increase the probability of NK cell infiltration in the TME by chemotaxis. High-throughput experimentation using our platform reduced the time and effort needed to optimize the culture condition by providing up to 14 identical vascularized tumor models for quantitative analysis in this study. The results obtained from experimental replications were consistent to allow statistical conclusions to be made, but we will need further investigation to identify complex molecular and cellular interactions under different conditions, which is beyond the scope of our study, for example, increases in the total vessel area and the number of junctions and differences in vascular permeability. EGM-2 supplemented with additional 10% FBS was used as the optimal cell culture medium throughout all experiments to successfully maintain perfusable blood vessels in the TME model.

We constructed a tumor vasculature model using six different CRC cell lines, which were chosen considering not only microsatellite stable (MSS)/microsatellite instability (MSI) subtype classification (35) but also recently identified CMS classification. Recent trends in studies regarding CRC underline that considering the CMS classification enhances our understanding of immune cell infiltration and immune responses in CRC (36). As the CMS classification categorizes CRC into four molecular subtypes with distinct biological characteristics, we expected different immune responses of the cellular components in the TME of various CRC cell lines in our model. CMS1 CRCs are called “immune activated” and recruit many active intratumoral immune cells toward the TME. On the other hand, CMS3 CRC cells are called “immune excluded” and have only certain intratumoral immune cells (37). According to our cytotoxicity assays using the tumor vasculature model, the high cytotoxicity activity of NK cells was observed in CMS1 CRC cells compared to other subtypes (**Figure 5C**). The size distribution of cancer cell clusters was also quantitatively analyzed to describe whether large cancer cell clusters in close contact with blood vessels provided more chances for NK cell extravasation, and no correlation was observed between them (**Supplementary Figure S2A–C** and **Figures 6A–C**). NK cell extravasation and cytotoxicity also did not correlate with vascular permeability. This can be interpreted as that NK cell extravasation is more induced by chemokines and their receptors expressed in cancer cells and NK cells, such as CXCL12/CXCR4 (38), than by the leakiness of the tumor vasculature. Further investigation will be required to study the recruitment of immune cells in the TME.

Our results confirmed that our platform provides a clinically meaningful understanding of the interaction between cancer and immune cells and can be used as a potential substitute for conventional cancer research models. Further studies using our model will explore tumor-immune system interactions with genetic analysis, since this preliminary study only relies on imaging-based analysis. Anticancer drug screening to either assess drug efficacy or improve the cytotoxic activity of NK cells is the next step of our research. The use of patient-derived cancer cells or genetically engineered immune cells can be considered as future work to validate the efficacy of immunotherapy including adoptive cell transfer.

In conclusion, we developed a tumor vasculature model using a high-throughput injection-molded microfluidic platform that can recapitulate key components of the TME. The platform that comprised 28 wells in a standardized format allowed us to perform high-throughput experiments and provided an effective method to define the optimal cell culture conditions for perfusable blood vessel formation. Vascular permeability coefficients and cancer cell cluster sizes were compared between six CRC cell lines, and as one of the possible applications of our tumor vasculature model, primary NK cells were used to demonstrate NK cell cytotoxicity assays based on different CMS of CRC cell lines. Intriguingly, the highest cytotoxic activity of NK cells was observed in CMS1 CRC cell lines, LoVo and SW48. Our platform has a wide range of potential applications, from studying immune-cancer cell interplays in the TME to drug screening for chemotherapy and immunotherapy.

## Data Availability Statement

The original contributions presented in the study are included in the article/**Supplementary Material**, further inquiries can be directed to the corresponding author.

## Ethics Statement

The use of human primary NK cells was approved by Institutional Review Board in 108 Samsung Medical Center (No. SMC 2018-02-102).

## Author Contributions

JS and HC conceptualized the study, performed the experiments, carried out the implementation and visualization, and wrote the manuscript. SK contributed to NK cell preparation and writing of the manuscript. DP, HK, and JY contributed to experimental work. YT was involved in data analysis. NJ and DC supervised the study. All authors contributed to the article and approved the submitted version.

## Funding

This work was supported by the National Research Foundation of Korea(NRF) grant funded by the Korea government(MSIT) (No.2019R1A4A2001651 and No.2021R1A3B1077481).

## Conflict of Interest

NJ is a founder and CTO of Qureator which has commercialized the chips used in this work.

The remaining authors declare that the research was conducted in the absence of any commercial or financial relationships that could be construed as a potential conflict of interest.

## Publisher’s Note

All claims expressed in this article are solely those of the authors and do not necessarily represent those of their affiliated organizations, or those of the publisher, the editors and the reviewers. Any product that may be evaluated in this article, or claim that may be made by its manufacturer, is not guaranteed or endorsed by the publisher.

## Supplementary Material

Supplementary Video S1 NK cytotoxicity against HT29.

Supplementary Video S2 NK cytotoxicity against HT29 (cropped).

Supplementary Video S3 NK cytotoxicity against SW48.

Supplementary Video S4 NK cytotoxicity against SW48 (cropped).

Supplementary Video S5 NK cytotoxicity against LoVo.

Supplementary Video S6 NK cytotoxicity against LoVo (cropped).

## References

[1] Arruebo M Vilaboa N Saez-Gutierrez B Lambea J Tres A Valladares M. Assessment of the Evolution of Cancer Treatment Therapies. Cancers (Basel) (2011) 3(3):3279–330. doi: 10.3390/cancers3033279 PMC3759197 24212956

[2] Waldman AD Fritz JM Lenardo MJ. A Guide to Cancer Immunotherapy: From T Cell Basic Science to Clinical Practice. Nat Rev Immunol (2020) 20(11):651–68. doi: 10.1038/s41577-020-0306-5 PMC7238960 32433532

[3] Lim WA June CH. The Principles of Engineering Immune Cells to Treat Cancer. Cell (2017) 168(4):724–40. doi: 10.1016/j.cell.2017.01.016 PMC5553442 28187291

[4] Liu E Marin D Banerjee P Macapinlac HA Thompson P Basar R. Use of CAR-Transduced Natural Killer Cells in CD19-Positive Lymphoid Tumors. N Engl J Med (2020) 382(6):545–53. doi: 10.1056/NEJMoa1910607 PMC7101242 32023374

[5] Shoemaker RH. The NCI60 Human Tumour Cell Line Anticancer Drug Screen. Nat Rev Cancer (2006) 6(10):813–23. doi: 10.1038/nrc1951 16990858

[6] Siolas D Hannon GJ. Patient-Derived Tumor Xenografts: Transforming Clinical Samples Into Mouse Models. Cancer Res (2013) 73(17):5315–9. doi: 10.1158/0008-5472.CAN-13-1069 PMC3766500 23733750

[7] Hoarau-Vechot J Rafii A Touboul C Pasquier J. Halfway Between 2D and Animal Models: Are 3D Cultures the Ideal Tool to Study Cancer-Microenvironment Interactions? Int J Mol Sci (2018) 19(1):181. doi: 10.3390/ijms19010181 PMC5796130 29346265

[8] Jensen C Teng Y. Is It Time to Start Transitioning From 2D to 3D Cell Culture? Front Mol Biosci (2020) 7:33. doi: 10.3389/fmolb.2020.00033 32211418 PMC7067892

[9] Boucherit N Gorvel L Olive D. 3d Tumor Models and Their Use for the Testing of Immunotherapies. Front Immunol (2020) 11:603640. doi: 10.3389/fimmu.2020.603640 33362787 PMC7758240

[10] Neal JT Li X Zhu J Giangarra V Grzeskowiak CL Ju J. Organoid Modeling of the Tumor Immune Microenvironment. Cell (2018) 175(7):1972–88 e16. doi: 10.1016/j.cell.2018.11.021 30550791 PMC6656687

[11] Parlato S De Ninno A Molfetta R Toschi E Salerno D Mencattini A. 3d Microfluidic Model for Evaluating Immunotherapy Efficacy by Tracking Dendritic Cell Behaviour Toward Tumor Cells. Sci Rep (2017) 7(1):1093. doi: 10.1038/s41598-017-01013-x 28439087 PMC5430848

[12] Pavesi A Tan AT Koh S Chia A Colombo M Antonecchia E. A 3D Microfluidic Model for Preclinical Evaluation of TCR-Engineered T Cells Against Solid Tumors. JCI Insight (2017) 2(12):e.89762. doi: 10.1172/jci.insight.89762 PMC5472441 28614795

[13] Park D Son K Hwang Y Ko J Lee Y Doh J. High-Throughput Microfluidic 3D Cytotoxicity Assay for Cancer Immunotherapy (CACI-IMPACT Platform). Front Immunol (2019) 10:1133. doi: 10.3389/fimmu.2019.01133 31191524 PMC6546835

[14] Lugano R Ramachandran M Dimberg A. Tumor Angiogenesis: Causes, Consequences, Challenges and Opportunities. Cell Mol Life Sci (2020) 77(9):1745–70. doi: 10.1007/s00018-019-03351-7 PMC7190605 31690961

[15] Schaaf MB Garg AD Agostinis P. Defining the Role of the Tumor Vasculature in Antitumor Immunity and Immunotherapy. Cell Death Dis (2018) 9(2):115. doi: 10.1038/s41419-017-0061-0 29371595 PMC5833710

[16] Goel S Duda DG Xu L Munn LL Boucher Y Fukumura D. Normalization of the Vasculature for Treatment of Cancer and Other Diseases. Physiol Rev (2011) 91(3):1071–121. doi: 10.1152/physrev.00038.2010 PMC3258432 21742796

[17] Ribatti D Annese T Ruggieri S Tamma R Crivellato E. Limitations of Anti-Angiogenic Treatment of Tumors. Transl Oncol (2019) 12(7):981–6. doi: 10.1016/j.tranon.2019.04.022 PMC6529826 31121490

[18] Aung A Kumar V Theprungsirikul J Davey SK Varghese S. An Engineered Tumor-On-a-Chip Device With Breast Cancer–Immune Cell Interactions for Assessing T-Cell Recruitment. Cancer Res (2020) 80(2):263–75. doi: 10.1158/0008-5472.CAN-19-0342 PMC8545579 31744818

[19] Ayuso JM Rehman S Virumbrales-Munoz M McMinn PH Geiger P Fitzgerald C. Microfluidic Tumor-on-a-Chip Model to Evaluate the Role of Tumor Environmental Stress on NK Cell Exhaustion. Sci Adv (2021) 7(8):eabc2331. doi: 10.1126/sciadv.abc2331 33597234 PMC7888951

[20] Boussommier-Calleja A Atiyas Y Haase K Headley M Lewis C Kamm RD. The Effects of Monocytes on Tumor Cell Extravasation in a 3D Vascularized Microfluidic Model. Biomaterials (2019) 198:180–93. doi: 10.1016/j.biomaterials.2018.03.005 PMC6123301 29548546

[21] Liu Y Sakolish C Chen Z Phan DTT Bender RHF Hughes CCW. Human In Vitro Vascularized Micro-Organ and Micro-Tumor Models Are Reproducible Organ-on-a-Chip Platforms for Studies of Anticancer Drugs. Toxicology (2020) 445:152601. doi: 10.1016/j.tox.2020.152601 32980478 PMC7606810

[22] Guinney J Dienstmann R Wang X de Reyniès A Schlicker A Soneson C. The Consensus Molecular Subtypes of Colorectal Cancer. Nat Med (2015) 21(11):1350–6. doi: 10.1038/nm.3967 PMC4636487 26457759

[23] Thangaraj JL Phan M-TT Kweon S Kim J Lee J-M Hwang I. Expansion of Cytotoxic Natural Killer Cells in Multiple Myeloma Patients Using K562 Cells Expressing OX40 Ligand and Membrane-Bound IL-18 and IL-21. Cancer Immunol Immunother (2021) 1–13. doi: 10.1007/s00262-021-02982-9 34282497 PMC10991462

[24] Lee S Kang H Park D Yu J Koh SK Cho D. Modeling 3d Human Tumor Lymphatic Vessel Network Using High-Throughput Platform. Adv Biol (2021) 5(2):2000195. doi: 10.1002/adbi.202170021

[25] Lee Y Choi JW Yu J Park D Ha J Son K. Microfluidics Within a Well: An Injection-Molded Plastic Array 3D Culture Platform. Lab Chip (2018) 18(16):2433–40. doi: 10.1039/C8LC00336J 29999064

[26] Choi SYC Collins CC Gout PW Wang Y. Cancer-Generated Lactic Acid: A Regulatory, Immunosuppressive Metabolite? J Pathol (2013) 230(4):350–5. doi: 10.1002/path.4218 PMC3757307 23729358

[27] Barbera-Guillem E Nyhus JK Wolford CC Friece CR Sampsel JW. Vascular Endothelial Growth Factor Secretion by Tumor-Infiltrating Macrophages Essentially Supports Tumor Angiogenesis, and IgG Immune Complexes Potentiate the Process. Cancer Res (2002) 62(23):7042–9. 12460925

[28] Faes S Uldry E Planche A Santoro T Pythoud C Demartines N. Acidic pH Reduces VEGF-Mediated Endothelial Cell Responses by Downregulation of VEGFR-2; Relevance for Anti-Angiogenic Therapies. Oncotarget (2016) 7(52):86026–38. doi: 10.18632/oncotarget.13323 PMC5349894 27852069

[29] Ahmed D Eide PW Eilertsen IA Danielsen SA Eknæs M Hektoen M. Epigenetic and Genetic Features of 24 Colon Cancer Cell Lines. Oncogenesis (2013) 2(9):e71–e. doi: 10.1038/oncsis.2013.35 PMC3816225 24042735

[30] Lee H Kim S Chung M Kim JH Jeon NL. A Bioengineered Array of 3D Microvessels for Vascular Permeability Assay. Microvasc Res (2014) 91:90–8. doi: 10.1016/j.mvr.2013.12.001 24333621

[31] Waldhauer I Steinle A. NK Cells and Cancer Immunosurveillance. Oncogene (2008) 27(45):5932–43. doi: 10.1038/onc.2008.267 18836474

[32] Siemann DW. The Unique Characteristics of Tumor Vasculature and Preclinical Evidence for Its Selective Disruption by Tumor-Vascular Disrupting Agents. Cancer Treat Rev (2011) 37(1):63–74. doi: 10.1016/j.ctrv.2010.05.001 20570444 PMC2958232

[33] Zhang B Hu Y Pang Z. Modulating the Tumor Microenvironment to Enhance Tumor Nanomedicine Delivery. Front Pharmacol (2017) 8:952. doi: 10.3389/fphar.2017.00952 29311946 PMC5744178

[34] Huber V Camisaschi C Berzi A Ferro S Lugini L Triulzi T. Cancer Acidity: An Ultimate Frontier of Tumor Immune Escape and a Novel Target of Immunomodulation. Semin Cancer Biol (2017) 43:74–89. doi: 10.1016/j.semcancer.2017.03.001 28267587

[35] De Smedt L Lemahieu J Palmans S Govaere O Tousseyn T Van Cutsem E. Microsatellite Instable vs Stable Colon Carcinomas: Analysis of Tumour Heterogeneity, Inflammation and Angiogenesis. Br J Cancer (2015) 113(3):500–9. doi: 10.1038/bjc.2015.213 PMC4522625 26068398

[36] Okita A Takahashi S Ouchi K Inoue M Watanabe M Endo M. Consensus Molecular Subtypes Classification of Colorectal Cancer as a Predictive Factor for Chemotherapeutic Efficacy Against Metastatic Colorectal Cancer. Oncotarget (2018) 9(27):18698–711. doi: 10.18632/oncotarget.24617 PMC5922348 29721154

[37] Picard E Verschoor CP Ma GW Pawelec G. Relationships Between Immune Landscapes, Genetic Subtypes and Responses to Immunotherapy in Colorectal Cancer. Front Immunol (2020) 11:369. doi: 10.3389/fimmu.2020.00369 32210966 PMC7068608

[38] Susek KH Karvouni M Alici E Lundqvist A. The Role of CXC Chemokine Receptors 1–4 on Immune Cells in the Tumor Microenvironment. Front Immunol (2018) 9:2159. doi: 10.3389/fimmu.2018.02159 30319622 PMC6167945

