# Supplementary material for: High-Throughput 3D In Vitro Tumor Vasculature Model for Real-Time Monitoring of Immune Cell Infiltration and Cytotoxicity
Source: Front Immunol. 2021 Sep 24;12:733317. doi: 10.3389/fimmu.2021.733317 (PMC8500473; doi:10.3389/fimmu.2021.733317)
Supplement: Supplementary file 7 [file DataSheet_1.docx]

Supplementary Material

**Supplementary Table 1.** Molecular characteristics of CRC cell lines.

**
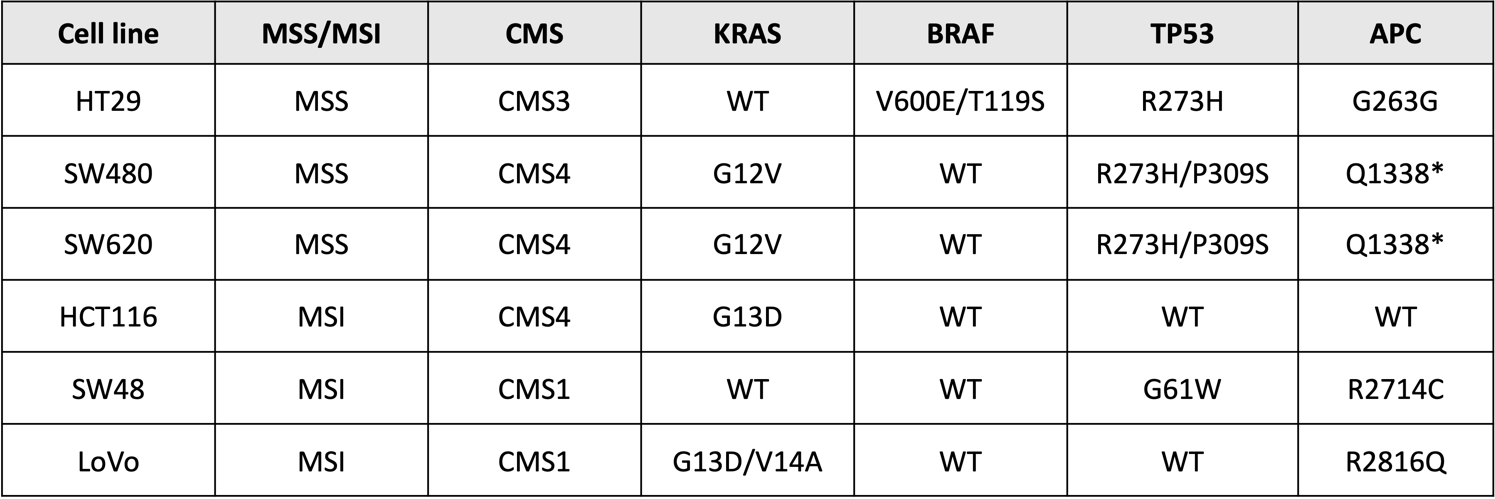
**


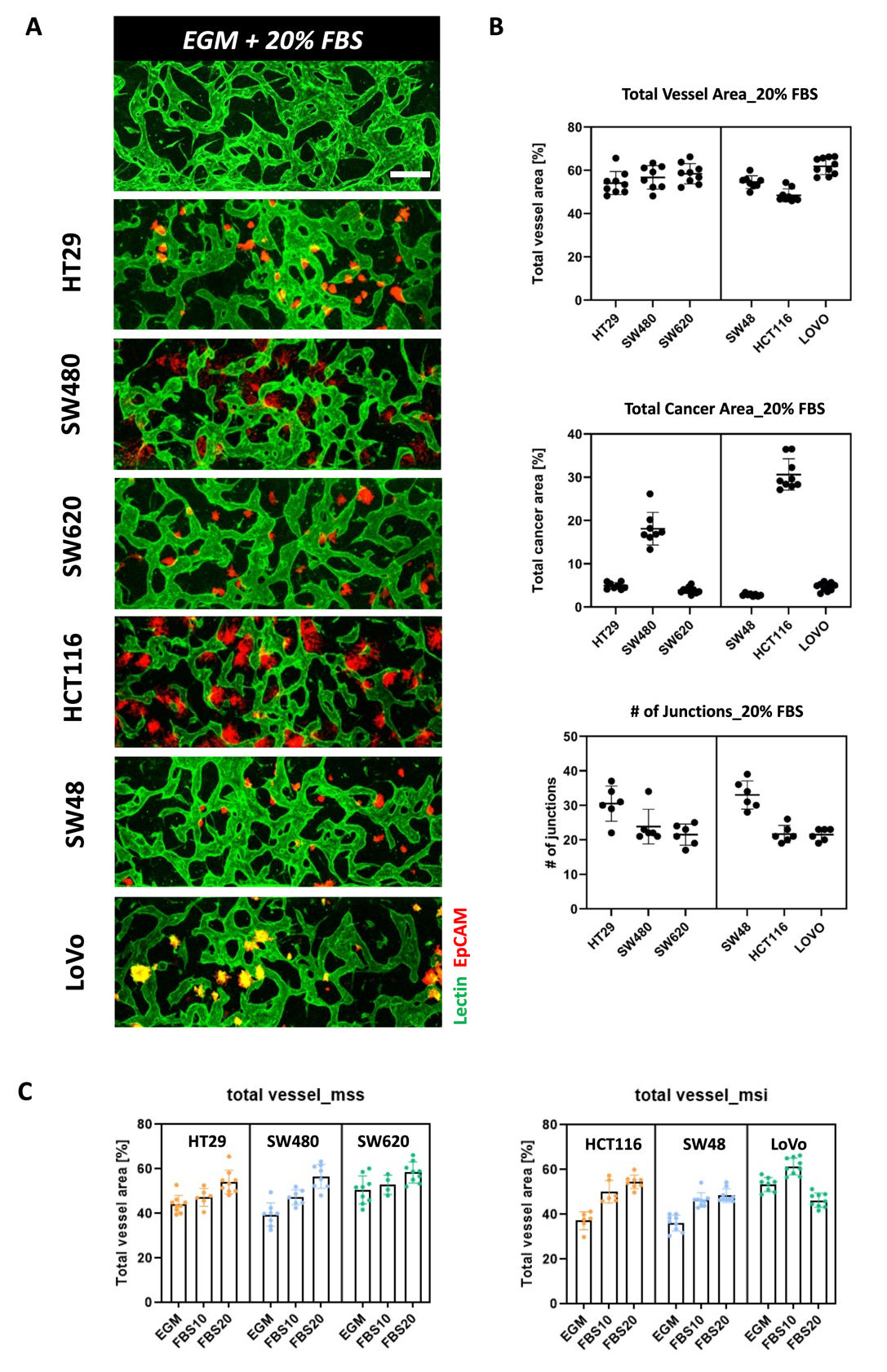


**Supplementary Figure 1.** Total vessel area and cancer cell cluster size under the condition with 20% FBS. (Related to Figure 2) **(A)** Confocal images of tumor vasculature (green) with various CRC cells (red). **(B)** Total vessel area, total cancer area, and the number of junctions of each tumor vasculature. **(C)** The comparison between EGM-2 and EGM-2 supplemented with 10% and 20% FBS according to MSS/MSI subtypes of CRC. All tumor vasculature showed a similar tendency (increasing total vessel area) (n = 6 to 10 for each CRC cell line). *p < 0.1; **p < 0.01; ***p < 0.001; ****p < 0.0001; ns, not significant. Scale bar = 200µm.


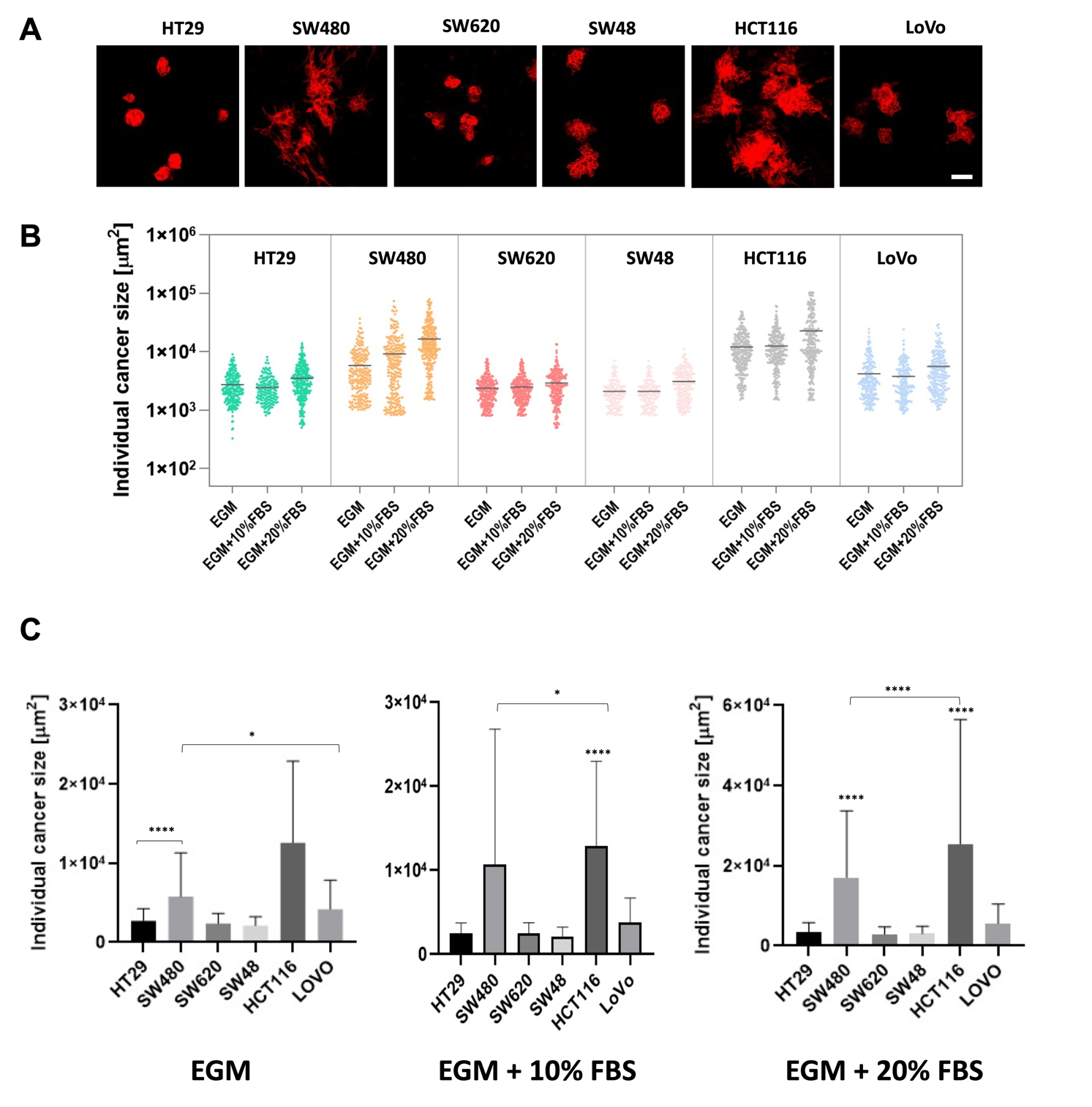


**Supplementary Figure 2.** The size of individual cancer cell clusters of each CRC cell line. **(A)** 3D formation of cancer cell clusters. HT29, SW620, and SW48 formed clusters in a spherical shape while HCT116 and SW480 formed clusters in a sparse star shape. **(B)** The size of individual cancer cell clusters formed in different cell culture medium and **(C)** their statistical graphs. *p < 0.1; **p < 0.01; ***p < 0.001; ****p < 0.0001; ns, not significant. Scale bar = 100µm.

**
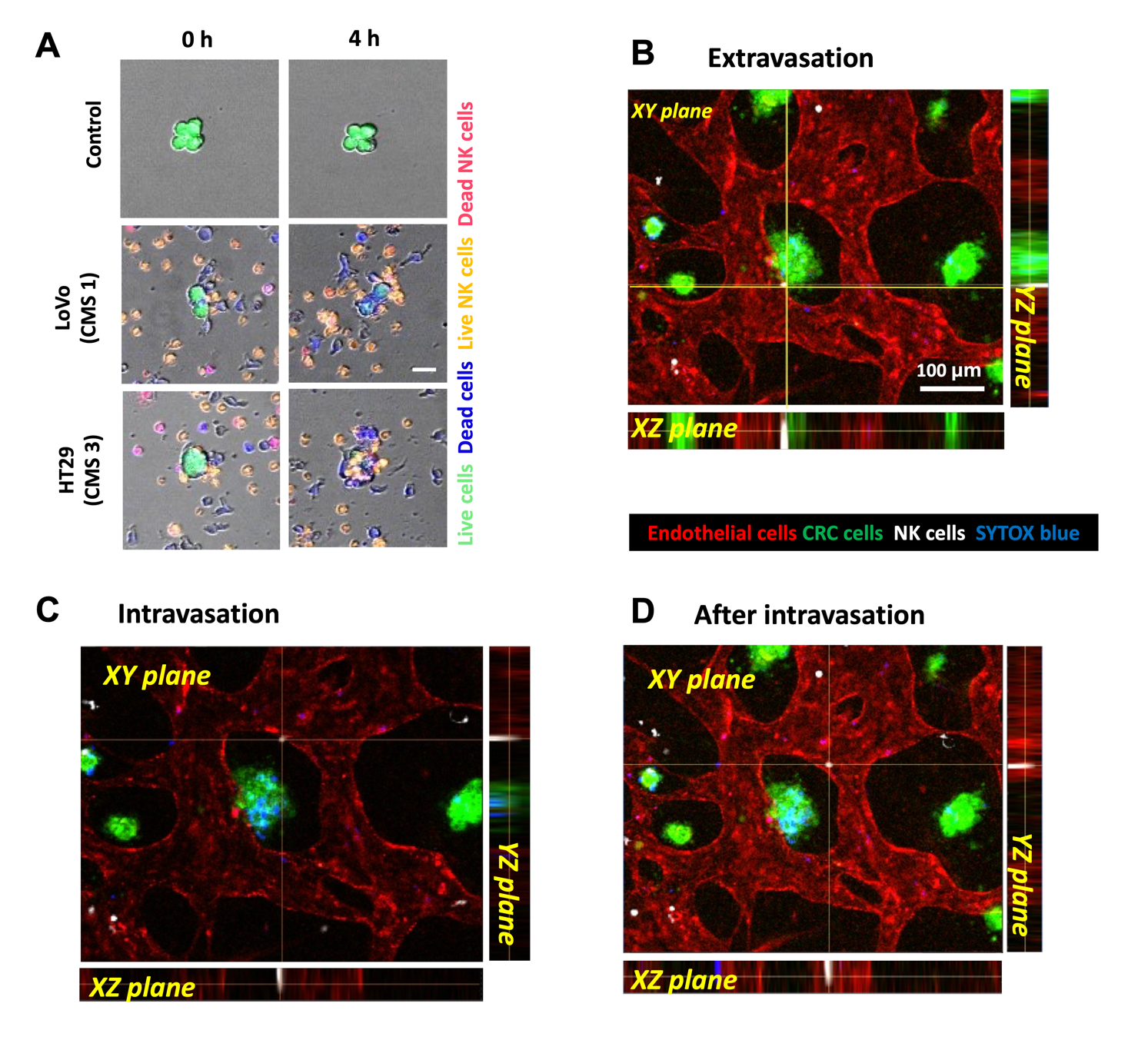
**

**Supplementary Figure 3.** NK cell cytotoxicity assays in 2D and 3D environments. **(A)** NK cell cytotoxicity assays performed in a conventional 2D cell culture. **(B to D)** 3D confocal images of NK cell extravasation and intravasation with the cross-section views in the X-Z plane and Y-Z plane.
